# Understanding the Role of Histidine in the GHSxG Acyltransferase Active Site Motif: Evidence for Histidine Stabilization of the Malonyl-Enzyme Intermediate

**DOI:** 10.1371/journal.pone.0109421

**Published:** 2014-10-06

**Authors:** Sean Poust, Isu Yoon, Paul D. Adams, Leonard Katz, Christopher J. Petzold, Jay D. Keasling

**Affiliations:** 1 Department of Chemical and Biomolecular Engineering, University of California, Berkeley, California, United States of America; 2 QB3 Institute, University of California, Berkeley, California, United States of America; 3 Synthetic Biology Engineering Research Center, Emeryville, California, United States of America; 4 Joint BioEnergy Institute, Emeryville, California, United States of America; 5 Physical Bioscience division, Lawrence Berkeley National Laboratory, Berkeley, California, United States of America; Institute of Enzymology of the Hungarian Academy of Science, Hungary

## Abstract

Acyltransferases determine which extender units are incorporated into polyketide and fatty acid products. The ping-pong acyltransferase mechanism utilizes a serine in a conserved GHSxG motif. However, the role of the conserved histidine in this motif is poorly understood. We observed that a histidine to alanine mutation (H640A) in the GHSxG motif of the malonyl-CoA specific yersiniabactin acyltransferase results in an approximately seven-fold higher hydrolysis rate over the wildtype enzyme, while retaining transacylation activity. We propose two possibilities for the reduction in hydrolysis rate: either H640 structurally stabilizes the protein by hydrogen bonding with a conserved asparagine in the ferredoxin-like subdomain of the protein, or a water-mediated hydrogen bond between H640 and the malonyl moiety stabilizes the malonyl-O-AT ester intermediate.

## Introduction

**Figure 1:**
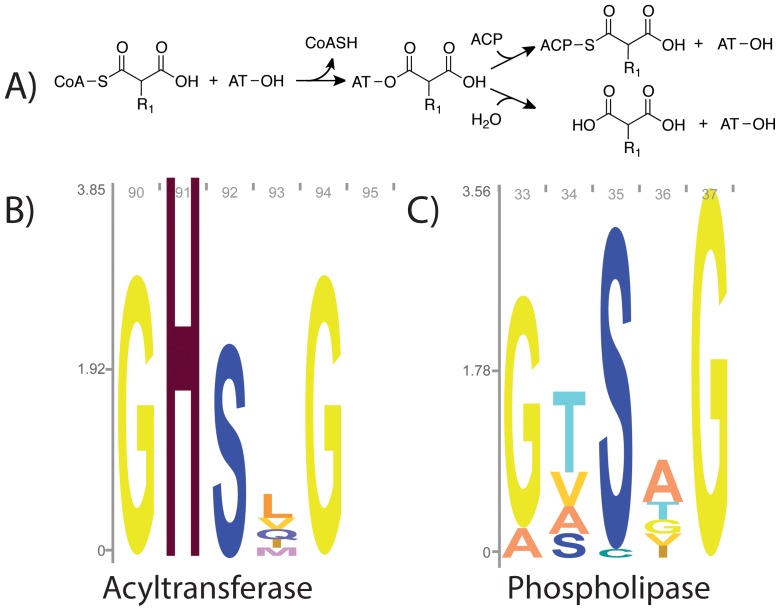
Acyltransferase reaction mechanism and sequence logos. A) AT reaction mechanism: ATs utilize a ping-pong mechanism to transfer acyl groups from CoA to ACP (for most ATs, R_1_ = H or CH_3_). Hydrolysis, shown in the bottom branch is a competing, unproductive reaction. B) Sequence logo for the motif containing the active site serine of pfam PF00698, which encompasses ATs from fatty acid and polyketide biosynthesis. C) Sequence logo for pfam PF01734, which are evolutionarily related phospholipases. Logos created using Skyline [12].

During fatty acid and polyketide biosynthesis, acyltransferases (ATs) catalyze transfer of the acyl group from malonyl-, methylmalonyl-, or other short chain acyl-CoAs to acyl carrier proteins (ACPs) using a serine-histidine catalytic dyad. The OH-group of serine acts as a nucleophile to attack the thioester bond of the acyl donor, forming a covalent acyl-*O*-ester intermediate. The acyl group is subsequently transferred to the ACP via a bi bi ping pong mechanism (Figure 1 A) [1].

For efficient transfer to the ACP, acyl-AT complexes must be stable in solution. However, ATs have been described as distant relatives of the α/β hydrolase superfamily [1], [2], and hydrolysis is an unproductive side reaction, effectively wasting the activated acyl-CoA substrate. The active sites for α/β hydrolases carry a GxSxG motif, whereas ATs possess the highly conserved GHSxG motif in the active site, suggesting that the histidine is important for acyl transfer. These conserved GHSxG and GxSxG motifs are illustrated in sequence logos from pfam PF00698 (Acyl_transf_1) and an evolutionarily related family from the same pfam clan, PF01734 (Patatin), which have phospholipase activity (Figure 1 B and C) [3]. The sequence logo for the AT family (Figure 1 B) shows that that the histidine in the GHSxG motif is highly conserved.

Dreier and coworkers proposed that the conserved histidine in the GHSxG motif could act as an alternative catalytic nucleophile responsible for the observed transacylation activity of an AT mutant in which the active site serine was changed to alanine [4]. This hypothesis was refuted by Szafranska and coworkers, who found the same serine to alanine mutant had no acyl transfer activity upon additional purification of the AT [5]. We sought to further investigate and clarify the role of the highly conserved histidine in acyl transfer reactions. Specifically, we examined three variants of the GHSxG motif in the malonyl-CoA specific yersiniabactin synthase AT domain [6]: S641A, H640A, and the double mutant H640A+S641A in the context of the full PKS module.

## Materials and Methods

His-tagged constructs were expressed in *Escherichia coli* BLR, and purified using Ni-NTA chromatography followed by anion exchange chromatography using a HiTRAP Q column (GE Healthcare). We observed that Ni-NTA chromatography alone was insufficient to remove background acyltransfer activity from negative controls (e.g. the double mutant S641A+H640A). For each variant, we measured steady-state rates of malonyl-CoA hydrolysis using a coupled fluorometric assay [7], [8], at physiological concentrations of malonyl-CoA [9]. This fluorometric coupled assay has been previously utilized to measure rates of acyl-CoA hydrolysis by ATs [8]. We also investigated the formation of malonyl-O-AT and the subsequent intraprotein transfer to form malonyl-S-ACP. We accomplished this by examining peptide acylation using high-resolution mass spectrometry after trypsin digestion of the protein following the *in vitro* loading/transfer reactions [10]. Detailed information on protocols is provided in the Methods S1.

## Results and Discussion

**Figure 2:**
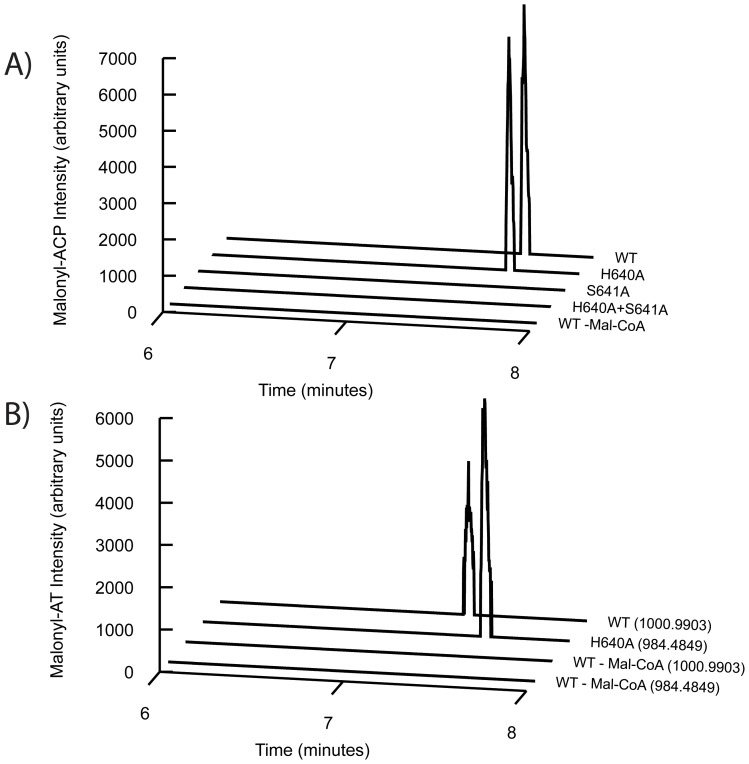
Acylation and transacylation activities of WT, H640A, S641A, and H640A+S641A. A) Transacylation activity as observed by high-resolution LC/QTOFMS. Data for the variants (WT, H640A, S641A, H640A+S641A) incubated with malonyl-CoA and a wildtype negative control without malonyl-CoA are shown (WT – Mal-CoA). B) Formation of malonyl-AT complex for wildtype (monoisotopic peptide m/z  =  1000.9903, z  =  4) and H640A (monoisotopic peptide m/z  =  984.4849, z  =  4) as observed by high-resolution LC/QTOFMS. Data for wildtype and H640A (WT, H640A) incubated with malonyl-CoA as well as a wildtype negative control without malonyl-CoA (WT – Mal-CoA) are shown. The mass for each chromatogram is shown in parenthesis to the right. Additional details on chromatogram preparation in the Methods S1.

Mutating H640 to alanine (mutant H640A) resulted in an approximately 7-fold increase in the malonyl-CoA hydrolysis rate over the wildtype enzyme (WT) rate (Table 1). Steady-state hydrolysis rates were measured at a concentration of 35 µM malonyl-CoA, which is the concentration of malonyl-CoA in exponentially growing *E. coli* as measured by Bennett and coworkers [9]. These values likely represent k_cat_ values, as the K_m_ for cognate acyl-CoA hydrolysis by other acyltransferases has been previously measured to be in the low uM range [8]. As expected, when the active site serine was mutated to an alanine, hydrolysis was not detectable (Table 1, S641A and S641A+H640A). Despite the increased rate of malonyl-CoA hydrolysis, the H640A mutant was still capable of transferring the malonyl moiety to the ACP when incubated with 35 µM malonyl-CoA. Loading/transfer reactions were quenched with 50% acetonitrile after 20 seconds and we detected similar amounts of malonyl-S-ACP (monoisotopic peptide *m/z*  =  1141.0562, *z*  =  4) for both wildtype and H640A (Figure 2 A). As with the hydrolysis assay, the S641A and S641A+H640A mutants had no detectable transacylation ability. Additionally, we observed malonyl-O-AT complex formation for both the wildtype (monoisotopic peptide *m/z*  =  1000.9903, *z*  =  4) and H640A (monoisotopic peptide *m/z*  =  984.4849, *z*  =  4) PKSs (Figure 2 B, additional details on chromatogram preparation in the Methods S1).

**Table 1:** Hydrolysis rates for yersiniabactin AT mutants at a concentration of 35 µM malonyl-CoA.

Variant	*v/[E]_o_* (min^−1^)	Fold increase over wildtype
Wildtype	0.39 +/− 0.09	1
H640A	3.01 +/− 0.32	7.7
S641A	n.d.	
H640A+S641A	n.d.	

n.d.  =  below limit of detection; error bars are the standard deviation of 3 replicates.

**Figure 3:**
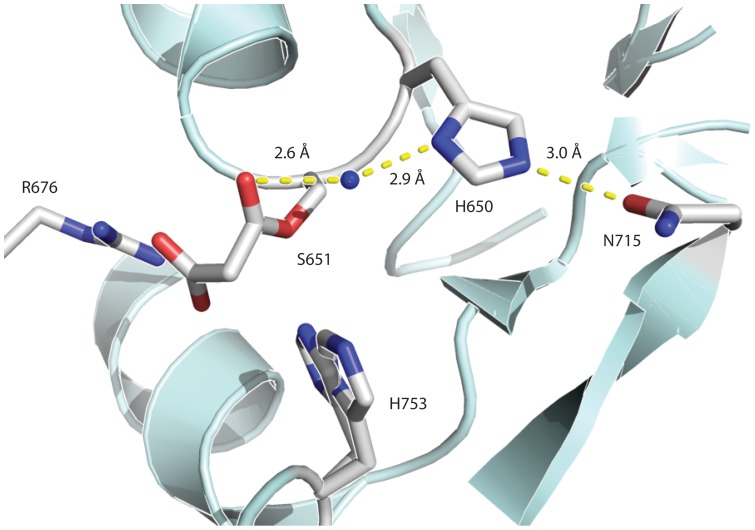
Crystal structure with malonate of the acyltransfserase from DynE8, an iterative type I PKS. Residues involved in catalysis are labeled and shown as sticks. The hydrogen bonding water highlighted in the text is shown as a sphere. Figure 3 was prepared using Pymol from the PDB entry 4AMP [11].

The question immediately arises as to why the removal of H640 increases the rate of hydrolysis. To address this issue, we examined the structure of the AT from DynE8, which has a covalently bound malonate (Figure 3) [11]. DynE8 is an iterative PKS involved in enediyne biosynthesis, and the DynE8 AT has 28% amino acid identity to the yersiniabactin AT. In this structure, the histidine in the GHSxG motif forms a hydrogen bond with a conserved asparagine in the ferredoxin-like subdomain (Figure 3). Through this hydrogen bond, the histidine may structurally stabilize the protein, reducing the hydrolysis rate. A water-mediated hydrogen bond between histidine in the GHSxG motif and the carbonyl oxygen in the AT ester bond is also present in the DynE8 structure (Figure 3), potentially stabilizing the malonyl-O-AT complex and slowing the rate of hydrolysis. We propose two possible mechanisms for this stabilization: either the water-mediated hydrogen bond favors the sp^2^ hybridization of the carbonyl oxygen in the malonyl ester bond over the sp^3^ hybridization of the tetrahedral transition state for hydrolysis; or the ordering of water provided by the hydrogen bonding network prevents water molecules from attacking the nearby carbonyl. We speculate that as the functional oxyanion hole forms upon the AT binding the ACP to facilitate transacylation (as proposed by Keatinge-Clay and coworkers) [1], the stabilizing hydrogen bonding interaction would be disrupted. Future crystallographic studies of AT active site mutants may elucidate the exact mechanism of stabilization.

In summary, we have proposed a stabilizing role of the conserved histidine in the GHSxG active site motif of the yersiniabactin synthase AT domain. To generalize, this suggests that ATs have evolved to protect acyl intermediates, functionally diverging from their α/β hydrolase relatives. Future work examining analogous histidine to alanine mutations in other AT domains would further support the role of the conserved histidine in stabilizing the malonyl-O-AT complex.

## Supporting Information

Methods S1 **Detailed materials and methods.**
(DOCX)
