# Supplementary material for: Understanding the Role of Histidine in the GHSxG Acyltransferase Active Site Motif: Evidence for Histidine Stabilization of the Malonyl-Enzyme Intermediate
Source: PLoS One. 2014 Oct 6;9(10):e109421. doi: 10.1371/journal.pone.0109421 (PMC4186864; doi:10.1371/journal.pone.0109421)
Supplement: Methods S1 — Detailed materials and methods. (DOCX) [file pone.0109421.s001.docx]

Supplementary Methods for

Title: Understanding the role of histidine in the GHSxG acyltransferase active site motif: evidence for histidine stabilization of the malonyl-enzyme intermediate

Authors: Sean Poust ^1^, Isu Yoon ^1^, Paul D. Adams ^4,5^, Leonard Katz ^3,4^, Christopher J. Petzold ^4,5^, and Jay D. Keasling ^*,1,2,3,4,5^

Affiliations:

^1^Department of Chemical and Biomolecular Engineering, University of California, Berkeley, CA 94270, USA

^2^QB3 Institute, University of California, Berkeley, CA 94270, USA

^3^Synthetic Biology Engineering Research Center, 5885 Hollis Street, Emeryville, CA 94608, USA

^4^Joint BioEnergy Institute, 5885 Hollis Street, Emeryville, CA 94608, USA

^5^Physical Bioscience division, Lawrence Berkeley National Laboratory, Berkeley, CA 94270, USA

*Corresponding author, email: keasling@berkeley.edu

­­

**Detailed Materials and Methods**

Reagents and Chemicals

Malonyl CoA, β-NAD+, NADH, α-ketoglutarate dehydrogenase (porcine heart) (αKGDH), α-ketoglutaric acid, thiamine pyrophosphate (TPP), TCEP and trypsin were purchased from Sigma (St. Louis, MO). HisPur Ni-NTA affinity resin was from Thermo. The Hi-Trap Q anion exchange column was from GE Healthcare. All primers were from IDT.

Cloning: The plasmid pSP8, expressing the wildtype yersiniabactin PKS was cloned by PCR amplification of the DNA sequence encoding amino acids 1-1895 (the PKS fragment) of HMWP1[1] using the forward primer 5’ – caaca**ccatgg**ataacttgcgcttc - 3’ (NcoI site in bold) and the reverse primer 5’ - caacac**ctcgag**cgcctctggcttagaacaga - 3’ (XhoI site in bold) using pET - 28 HMWP1 as a template.[2] The PCR amplicon was then cloned into the NcoI-XhoI sites of pET28b, encoding a C-terminally his-tagged yersiniabactin PKS. The sequence of the resulting pSP8 plasmid is publicly available on the JBEI public registry (public-registry.jbei.org, Part ID: JPUB_003691). Using pSP8 as a template, the S641A mutation was constructed by performing nested PCR encoding the mutation using the primer pairs CAACAC**CATATG**ATCGTCGCCTCGCTGCCC/CAAATTCACCGAC***GGC***ATGCCCAATGGCGAAGTCTGGC (NdeI site in bold and S641A mutation in bold italics) and CATTGGGCAT***GCC***GTCGGTGAATTTGCCGCTGCCG/CAACAC**GAATTC**CTGATACAGCACTTCCACGCCG (XhoI site in bold and S641A mutation in bold italics) to construct a PCR fragment with flanking NdeI-EcoRI sites, encoding a S641A mutation in the PKS sequence between the unique NdeI-EcoRI sites in pSP8. Primers were designed using j5.[3] This PCR fragment was then cloned into the unique NdeI-EcoRI sites in pSP8. To construct the mutations for the H640A and H640A+S641A mutants, Kunkel mutagenesis was performed as described[4] using the following antisense primers H640A: 5’ - caaattcaccgacgga***agc***cccaatggcgaagtctg - 3’. H6540A+S641A 5’ - ggcaaattcaccgac***ggcagc***cccaatggcgaagtctg - 3’ (sequence encoding mutations in bold italics). All constructs were sequenced verified by Quintara Biosciences (Richmond, CA).

Purification: An E. coli BLR strain harboring acyl transferase mutants were grown in 1 L 2xYT medium supplemented with appropriate antibiotics at 37°C until the OD600 reached 0.7-0.9. The cultures were then cooled to 18°C and induced with 75 µM isopropyl-β-D-galactopyranoside for 18 h. The cells were harvested by centrifugation (10000 x g, 5 min) and resuspended in lysis/wash buffer (25 mM Tris pH 8, 500 mM NaCl, 20 mM imidizole). The cells were lysed by sonication and cellular debris was removed by centrifugation (10000 x g, 10 min, 4°C). The supernatant was poured through a fritted column with 5 mL Nickel-NTA agarose resin twice, washed with 10 resin volumes of lysis/wash buffer (4°C), and eluted with 10 mL of elution buffer (25 mM Tris, pH 8, 150 mM imidazole, 4°C). The eluted protein was then applied to a HiTRAP Q anion exchange column (GE Healthcare), washed with 10 resin volumes of start buffer (25 mM Tris pH 8), and eluted at approximately 375 mM NaCl. The eluted protein was concentrated using an Amicon Ultra-15 Centrifugal Filter, 100K device (Millipore). Glycerol was added to the concentrated protein to 8% and was flash frozen in liquid nitrogen and stored at -80°C. The presence of holo-ACP was verified by proteomics. Protein concentrations were determined using Quick Start 1x Bradford Dye Reagent (Bio-Rad) using BSA as a standard.

Hydrolysis assay: Hydrolysis assays were based on a fluorometric transacylase assay described previously.[5] Hydrolysis assays were performed as described previously, using a Synergy H4 Hybrid Multi-Mode Microplate Reader (BioTek), except that EDTA and BSA were omitted from the assay mixture.[6] Final assay concentrations were: 50 mM sodium phosphate, pH 7.4, 10% glycerol, 1 mM TCEP, 0.4 mU/μL αKGDH, 0.4 mM NAD+, 0.4 mM TPP, and 2 mM α-ketoglutaric acid. The concentration of yersiniabactin PKS variants was 1 μM and the concentration of malonyl-CoA was 35 μM.[7] NADH was used to generate a standard curve. All reactions were run in triplicate.

Proteomics-based acylation/transacylation assay: Protein variants at a concentration of 1 µM were incubated with 35 µM malonyl-CoA in 100 mM ammonium bicarbonate, pH 7.8, and 1 mM TCEP for 20 seconds. The addition of malonyl-CoA was used to start the reaction. Reactions were quenched with 50% acetonitrile. The concentration of acetonitrile was diluted to 25% with water and proteins were digested with trypsin (0.5 µg/µl) at 37 °C for 4.5 hours at a ratio of 1:12.5 trypsin:protein. Samples were analyzed on an Agilent 1290 UHPLC system (Agilent Technologies, Santa Clara, CA) coupled to an Agilent 6550 Q-TOF mass spectrometer operating in MS1 mode. 0.25 microgram (µg) of protein was injected and separated on a Sigma Ascentis Peptide Express C-18 column (2.1 mm × 50 mm, 2.7 um particle size; Sigma-Aldrich, St. Louis, MO) at a flow rate of 400 µl/min with gradient conditions as follows: starting with 95% Buffer A (98% water, 2 % acetonitrile, 0.1 % formic acid) and 5% Buffer B (98% acetonitrile, 2% water, 0.1 % formic acid) for one minute, followed by an increase to 35% Buffer B over 5.5 min, followed by a rapid increase to 80% B and a flow rate of 600 µl/min in 1 min, where it was held for 4 min. The solvent composition was quickly ramped to 5% B and the flow rate reduced to 400 µl/min, where it was subsequently held for 2 min to allow the column to equilibrate for the next run. The peptides eluting from the column were ionized by using an Agilent Jet Stream source (sheath gas flow: 11 l/min, sheath gas temperature: 250 c, nozzle voltage: 1,000 v, nebulizing pressure: 35 psi, chamber voltage: 5000 V) operating in positive-ion mode. The data were acquired with MassHunter B.05.00 operating in MS1 mode within 300 m/z to 1400 m/z mass range.

Preparation of extracted ion chromatograms for Figure 2: A) Malonyl-ACP intensity peak intensity is plotted from extracted ion chromatograms of *m/z* = 1141.0562 +/- 0.02, which is the monoisotopic m/z of the tryptic peptide containing the phosphopantetheine arm of the ACP with a malonyl moiety attached (tryptic ACP peptide sequence: LSDPASLHPNQDLLQLGMDSLLFLELSSDIQHYLGVR). B) Malonyl-AT intensity peak intensity is plotted from extracted ion chromatograms of *m/z* =1000.9903 +/- 0.02 or *m/z* = 948.4849 +/- 0.02, which is the monoisotopic mass of the tryptic peptide containing the active site serine of the AT plus a malonyl moiety for wildtype and the H640A mutant, respectively (Wildtype AT tryptic peptide sequence: AEGLKPDFAIGHSVGEFAAAVVCGHYTIEQVMPLVCR).

**References**

1. Miller DA, Luo L, Hillson N, Keating TA, Walsh CT (2002) Yersiniabactin Synthetase: A Four-Protein Assembly Line Producing the Nonribosomal Peptide/Polyketide Hybrid Siderophore of Yersinia pestis. Chem Biol 9: 333-344.

2. Suo Z, Chen H, Walsh CT (2000) Acyl-CoA hydrolysis by the high molecular weight protein 1 subunit of yersiniabactin synthetase: Mutational evidence for a cascade of four acyl-enzyme intermediates during hydrolytic editing. Proc Natl Acad Sci 97: 14188-14193.

3. Hillson NJ, Rosengarten RD, Keasling JD (2011) j5 DNA Assembly Design Automation Software. ACS Synth Biol 1: 14-21.

4. Linshiz G, Stawski N, Goyal G, Bi C, Poust S, et al. (2014) PR-PR: Cross-Platform Laboratory Automation System. ACS Synth Biol.

5. Molnos J, Gardiner R, Dale GE, Lange R (2003) A continuous coupled enzyme assay for bacterial malonyl–CoA:acyl carrier protein transacylase (FabD). Anal Biochem 319: 171-176.

6. Dunn BJ, Cane DE, Khosla C (2013) Mechanism and Specificity of an Acyltransferase Domain from a Modular Polyketide Synthase. Biochemistry 52: 1839-1841.

7. Bennett BD, Kimball EH, Gao M, Osterhout R, Van Dien SJ, et al. (2009) Absolute metabolite concentrations and implied enzyme active site occupancy in Escherichia coli. Nat Chem Biol 5: 593-599.
